# Are the Symptoms of an NSAID-Induced Ulcer Truly Milder Than Those of an Ordinary Ulcer?

**DOI:** 10.1155/2017/4653250

**Published:** 2017-08-27

**Authors:** Toshihiko Tomita, Sumire Mori, Katsuyuki Tozawa, Eitatsu Arai, Nobuo Tano, Hideo Oka, Yongmin Kim, Takashi Abe, Yoshio Ohda, Tadayuki Oshima, Hirokazu Fukui, Jiro Watari, Hiroto Miwa

**Affiliations:** ^1^Division of Gastroenterology, Department of Internal Medicine, Hyogo College of Medicine, Hyogo, Japan; ^2^Department of Internal Medicine, Nishinomiya Kyoritsu Neurosurgical Hospital, Hyogo, Japan; ^3^Department of Internal Medicine, Kyoritsu Hospital, Hyogo, Japan; ^4^Department of Internal Medicine, Amagasaki Central Hospital, Hyogo, Japan; ^5^Division of Gastroenterology, Takarazuka Municipal Hospital, Hyogo, Japan

## Abstract

**Objective:**

The percentage of patients with nonsteroidal anti-inflammatory drugs (NSAIDs) and low-dose aspirin- (LDA-) induced ulcers who complain of gastrointestinal symptoms has generally been considered to be low. The aim of this study was to examine and compare the symptoms and quality of life (QOL) at peptic ulcer onset.

**Methods:**

This study involved 200 patients who were confirmed by endoscopy to be in the acute stage of gastroduodenal ulcer (A1-H1). Patients completed a self-administered questionnaire (Global Overall Symptom score and SF-8) at ulcer onset, and data were compared between NSAIDs/LDA ulcers and non-NSAIDs/LDA ulcers.

**Results:**

The upper gastrointestinal symptoms score was significantly lower for patients using LDA only (20.5 ± 9.4 in the nonusing group, 19.6 ± 8.6 in the NSAIDs-only group, 16.7 ± 11.6 in the LDA-only group, and 18.5 ± 7.2 in the NSAIDs/LDA group, *P* < 0.05). The QOL score (physical summary) was significantly lower in the NSAID group (42.1 ± 9.9) than in the nonusing group (47.6 ± 7.6) (*P* < 0.05). Patients' characteristics showed no significant differences among the groups, with the exception of age.

**Conclusion:**

The severity of upper abdominal symptoms at peptic ulcer onset was similar between NSAID users and nonusers.

## 1. Introduction

There has been a recent increase in the use of nonsteroidal anti-inflammatory drugs (NSAIDs) and low-dose aspirin (LDA), both of which are risk factors for peptic ulcers and upper gastrointestinal bleeding with prolonged administration [1–12]. This increased use of NSAIDs and LDA is particularly notable in the elderly [13], where in many cases the drugs are used for the treatment of underlying disease and cannot readily be discontinued [14, 15]. Recent Japanese clinical practice guidelines for the treatment of peptic ulcer are encouraging greater awareness of the potential for drug-induced peptic ulcer [16].

Several reports are available on the relationship between NSAID-induced ulcers and gastrointestinal symptoms; the percentage of patients with NSAID-induced ulcers who complain of gastrointestinal symptoms has generally been considered to be low [7, 17–21]. However, most of the reported research has failed to include control groups of patients who were not using NSAIDs or LDA, so no clear evidence has been gathered on whether gastric and duodenal ulcer symptoms are truly milder for NSAID-induced and LDA-induced ulcers.

Therefore, the effects of NSAID and/or LDA use on gastrointestinal symptoms when ulcers developed were investigated. In the present research, gastric and duodenal ulcers were identified by esophagogastroduodenoscopy (EGD). Patients with confirmed ulcers were then asked to complete questionnaires on their gastrointestinal symptoms and on health-related quality of life (QOL).

## 2. Patients and Methods

### 2.1. Patients

The subjects were 200 patients who visited our clinic or a related facility between January 2012 and December 2015, underwent EGD, and were consecutively diagnosed with acute peptic ulcer (stage A1-H1).

Patients who participated in this research were required to satisfy all of the following inclusion criteria: (1) male or female outpatient at least 20 years of age; (2) acute peptic or duodenal ulcer (stage A1-H1) confirmed by EGD; (3) able and willing to complete self-administered questionnaires for this research; and (4) provided written informed consent to participate in this research. Exclusion criteria were as follows: (1) gastrointestinal bleeding or warning symptoms of gastrointestinal bleeding at least 4 weeks previously (but no more than 8 weeks previously); (2) complications that might affect this research (malignant tumor, history of gastric resection, or other such surgery that might affect this research); or (3) considered unsuitable for participation by the physician in charge.

### 2.2. Study Design

The process began with EGD, after which patients with confirmed active peptic ulcers (A1-H1) were provided with information for informed consent and asked to provide their written informed consent to participate in this research. The Sakita classification was used for peptic ulcer staging [22]. Patients completed a self-administered questionnaire to score their gastrointestinal symptoms at ulcer onset. At the same time, the patients were also asked to use a health-related QOL questionnaire to score their condition at ulcer onset. Gastrointestinal symptoms were evaluated with the Global Overall Symptom (GOS) scale [23], and the Short-Form 8 Health Survey (SF-8) was used for health-related QOL [24]. GOS was defined as eight items to validate the dyspeptic symptoms and has been used in clinical studies to evaluate dyspeptic symptoms and treatment efficacy [25, 26]. We measured the severity of eight symptoms (epigastric pain, heartburn, regurgitation, postprandial fullness, nausea/vomiting, belching, early satiety, and bloating) on a 7-point Likert scale (1 = no problem [no symptoms]; 2 = minimal problem [can be easily ignored without effort]; 3 = mild problem [can be ignored with effort]; 4 = moderate problem [cannot be ignored but does not influence daily activities]; 5 = moderately severe problem [cannot be ignored and occasionally limits daily activities]; 6 = severe problem [cannot be ignored and often limits concentration on daily activities]; and 7 = very severe problem [cannot be ignored, markedly limits daily activities and often requires rest]).

The completed GOS was collected by the investigators who were not allowed to change any outcomes reported by the patients. Patients were excluded from consideration if they failed to answer even one question on the questionnaires.

Baseline patient characteristics were also assessed, including ordinary factors (sex, age, height, weight, and medical history), smoker/nonsmoker, drinker/nondrinker, NSAID user/nonuser (drug name, dose, and duration of use), LDA user/nonuser (drug name, dose, and duration of use), and peptic ulcer drug user/nonuser (drug name, dose, and duration of use). We performed all experiments in accordance with human ethics regulations (Hyogo College of Medicine: number 970). The trial was conducted according to the principles governing human research in the Declaration of Helsinki. All authors had access to the study data and reviewed and approved the final manuscript.

### 2.3. Outcome Measures

The primary endpoint was differences in gastrointestinal symptom scores and health-related QOL scores between users and nonusers of NSAIDs and LDA at peptic ulcer onset, compared with the following patient groups: NSAID-only, LDA-only, NSAIDs + LDA, and nonusing (neither NSAIDs nor LDA). The secondary endpoint was to evaluate the correlation between age and severity of upper abdominal symptoms at onset.

### 2.4. Statistical Analysis

All results were expressed as mean ± standard deviation (SD). Characteristics of enrolled patients were analyzed by Fisher's exact test, and only sex was analyzed by chi-square test. Upper abdominal symptom score and HRQOL score were determined using unpaired *t*-test. Correlation analysis was performed with Spearman's rank correlation coefficient (*rs*). Significance was defined as a value of *P* < 0.05.

## 3. Results

### 3.1. Characteristics of Enrolled Patients

**Figure 1:**
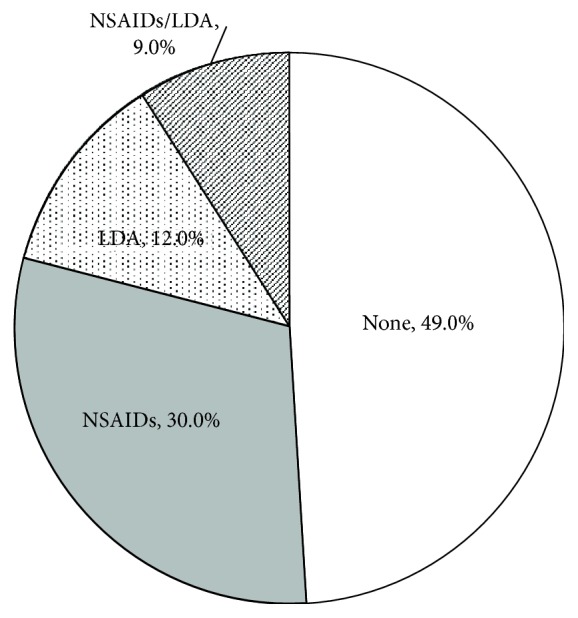
Characteristics of enrolled patients. NSAIDs: nonsteroidal anti-inflammatory drugs; LDA: low-dose aspirin.

The 174 analyzed subjects consisted of 106 men and 68 women, with a mean age of 64.3 ± 16 years. There were 108 patients with gastric ulcer, 43 with duodenal ulcer, and 23 with gastroduodenal ulcer. The patients who used neither NSAIDs nor LDA accounted for 49.0% (85 patients). The NSAID-only group included 52 patients (30.0%), the LDA-only group included 21 patients (12.0%), and the NSAID + LDA group had 16 patients (9.0%) (Figure 1). There were no significant differences among the groups in the patients' characteristics, with the exception of age (significantly higher in the LDA-only group than in the NSAID-only group and the nonusing group) (Table 1).

### 3.2. Upper Abdominal Symptom Score and Use or Nonuse of NSAIDs/LDA

**Figure 2:**
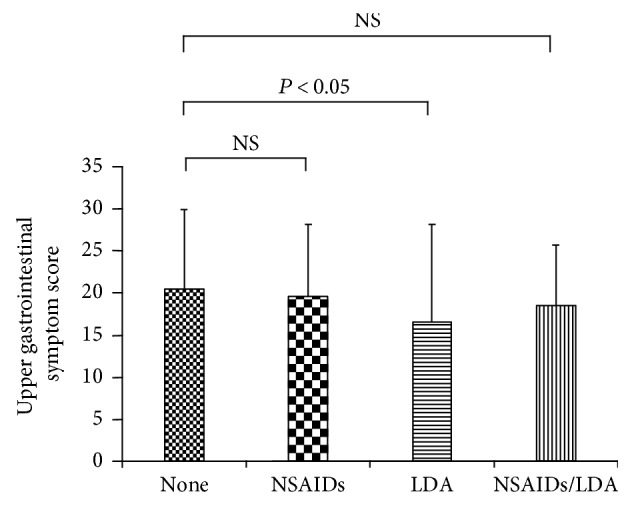
Upper abdominal symptom score and use of NSAIDs/LDA. NSAIDs: nonsteroidal anti-inflammatory drugs; LDA: low-dose aspirin.

**Figure 3:**
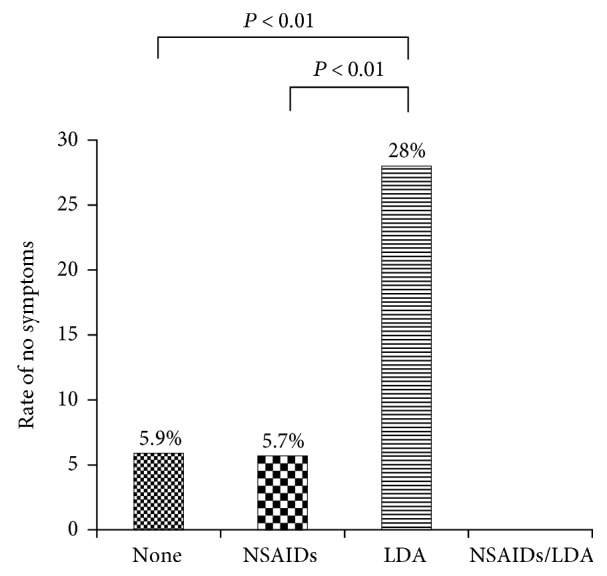
The rate of no symptoms at ulcer onset (GOS scale 8). GOS scale: Global Overall Symptom scale; NSAIDs: nonsteroidal anti-inflammatory drugs; LDA: low-dose aspirin.

The upper gastrointestinal symptom score was significantly lower in the LDA-only group (20.5 ± 9.4 in the nonusing group, 19.6 ± 8.6 in the NSAID-only group, 16.7 ± 11.6 in the LDA-only group, and 18.5 ± 7.2 in the NSAID + LDA group, *P* < 0.05, Figure 2). Symptoms were completely absent at ulcer onset in 5.9% of patients with gastroduodenal ulcers who were using neither NSAIDs nor LDA (NSAID/LDA nonusing group, 5/85 subjects) (Figure 3).

### 3.3. HRQOL Score and Use or Nonuse of NSAIDs/LDA

**Figure 4:**
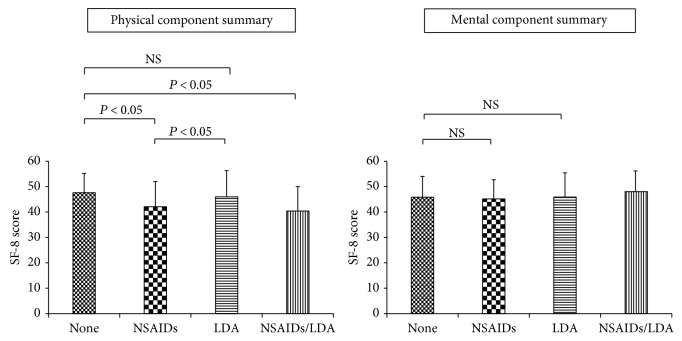
HRQOL score and use of NSAIDs/LDA. HRQOL: health-related quality of life; NSAIDs: nonsteroidal anti-inflammatory drugs; LDA: low-dose aspirin; SF-8: Short-Form 8 Health Survey.

The physical component summary score was significantly lower in the NSAID group than that in the nonusing group or the LDA group (47.6 ± 7.6 in the nonusing group, 42.1 ± 9.9 in the NSAID-only group, 46.0 ± 10.1 in the LDA-only group, and 40.4 ± 9.6 in the NSAID/LDA group, *P* < 0.05). The mental component summary score was 46.1 ± 8.2 in the nonusing group, 45.4 ± 7.6 in the NSAID-only group, 46.0 ± 9.5 in the LDA-only group, and 48.2 ± 8.3 in the NSAID/LDA group, with no significant difference among the four groups (Figure 4).

Among nonusing patients, the physical QOL score was 49.5 ± 6.7 for patients who had no symptoms at ulcer onset and 47.4 ± 7.6 for patients who had such symptoms, with no significant difference between the two groups. The psychological summary score for these patients was 48.9 ± 9.5 in patients who had no symptoms at ulcer onset, and 45.9 ± 8.2 for patients who had such symptoms, with no significant difference between the two groups.

### 3.4. Correlation between Age and Severity of Upper Abdominal Symptoms at Onset

**Figure 5:**
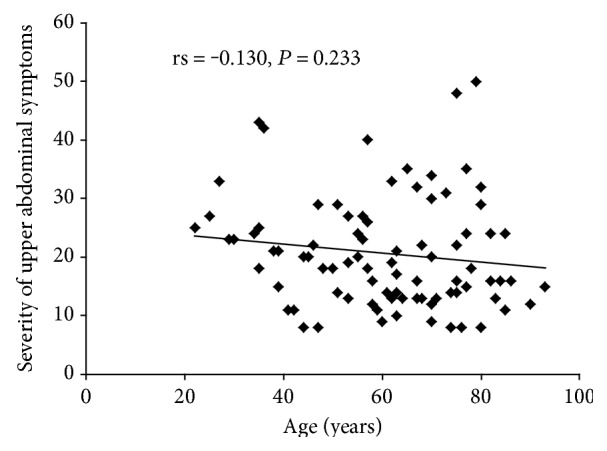
Correlation between age and severity of upper abdominal symptoms at onset.

The 85 patients in the nonusing group tended to show an inverse relationship between age and severity of upper abdominal symptoms at onset, but that relationship was not significant (rs = −0.130, *P* = 0.233) (Figure 5).

## 4. Discussion

This study is the first multicenter, observational study to examine and compare symptoms and QOL at ulcer onset between NSAID/LDA ulcers and non-NSAID/LDA ulcers in patients in the acute stage of peptic ulcer (A1-H1) as confirmed by EGD. Previous reports have assumed that NSAID-induced ulcers rarely cause symptoms, and that those symptoms develop suddenly [7, 17, 18]. A survey of gastrointestinal symptoms was conducted in about 1000 arthritis patients who were able to undergo endoscopy and were treated with NSAIDs for at least 3 months [17]. Of the subjects who developed a gastrointestinal mucous membrane disorder during treatment with NSAIDs, about half reported no symptoms at all. When the Lanza score, which assesses the extent of damage in gastric mucosal lesions, was used to evaluate patient symptoms, approximately 60% of patients with gastric mucosal damage reported no symptoms [18]. Because of such findings, asymptomatic gastrointestinal disorders have generally been considered characteristic of NSAID-induced gastrointestinal injury. The findings suggested that the analgesic effects of NSAIDs might have contributed to the asymptomatic nature of these NSAID-induced ulcers by relieving upper abdominal symptoms. However, because these studies did not include a control group of patients who were not taking NSAIDs, no conclusions could be drawn.

An association has also been reported between LDA, upper abdominal symptoms, and gastric mucous membrane disorders. EGD was conducted in 187 patients who were taking aspirin, and ulcers and erosions were assessed. Erosions were found in more than 60% of patients, and gastroduodenal ulcers were found in approximately 11%. However, investigation of the association between gastrointestinal symptoms and the presence or absence of ulcers showed that a high proportion (at least 50%) of patients were asymptomatic even when ulcers were present [7]. No significant difference was reported between the groups of patients with and without ulcers. This indicates that it is problematic to attempt to determine the presence of NSAID-induced or aspirin-induced ulcers simply from gastrointestinal symptoms. Such findings suggest that patients with drug-induced ulcers may show few symptoms, and that such symptoms may appear suddenly. However, that research also lacked a non-LDA-taking control group, so the findings cannot be considered scientifically proven. In the present research to investigate the symptoms of NSAID-induced and LDA-induced ulcers, ulcer symptoms were compared between an NSAID/LDA group and a non-NSAID/LDA group. That comparison showed clear symptoms in the NSAID/LDA group, which was interesting, given the common wisdom that such ulcers in such patients are generally asymptomatic.

In this study, the recall study method was used to investigate symptoms at the onset of NSAID-induced ulcers. Most ulcers resolve spontaneously, so for ulcers at stage H1 or above, ulcer onset was assumed within 1 month previously, and patients were asked to recall the most intense symptoms that they had experienced within that 1-month period. These symptoms at ulcer onset were then compared for the NSAID-induced ulcers and the ordinary ulcers. This method is limited by potential recall bias (the patient is responding to the questionnaire from memory and may not accurately record the actual symptoms) and by uncertain time of onset (the questionnaire is surveying the symptoms of ulcers for which the actual date of onset is unknown). However, such bias applies equally to both groups of patients, so results from this comparison between NSAID/LDA users and nonusers are expected to be meaningful.

In addition, in this research, each patient underwent endoscopy, and if the results clearly indicated the presence of a gastroduodenal ulcer, the patient was asked to complete a questionnaire on gastrointestinal symptom intensity at the time of ulcer onset. Gastroduodenal ulcers are often asymptomatic. However, the present research was designed to show the percentage of ulcer patients who reported no symptoms at ulcer onset. We believe the present findings provide important new insights, as 5.9% of patients with gastroduodenal ulcers were asymptomatic at the time of ulcer onset. QOL was also surveyed in NSAID/LDA nonusing patients. Although the difference was not significant, higher physical QOL scores were seen in patients who were asymptomatic at ulcer onset than in those patients who experienced symptoms. This higher physical QOL score provides scientific support for the intuitive assumption that QOL is improved by the absence of gastrointestinal symptoms.

The present research showed no difference in the intensity of gastrointestinal symptoms between NSAID and non-NSAID ulcers, but milder symptoms in LDA ulcers than in non-LDA ulcers. These findings are somewhat difficult to interpret. From the perspective of drug efficacy with regard to anti-inflammatory and analgesic effects, it seems unlikely that LDA ulcers would be associated with milder symptoms. Patient age appears to be one factor associated with the milder symptoms seen with LDA ulcers; there was a nonsignificant correlation between ulcer symptoms and patient age, with a tendency toward milder symptoms in older patients (data not shown). Data are also available from other studies that show a general decrease in symptoms with increasing age. In particular, an association has been reported between aging and gastrointestinal hypoesthesia in areas such as the esophagus, and the possibility of such effects cannot be ruled out in this case [2, 27–30]. With regard to upper abdominal symptoms, pain was significantly lower in the LDA-using group than in either the nonusing group or the NSAID group. No significant difference was noted in other symptoms such as indigestion and heartburn.

In this research, health-related QOL was also investigated. The correlation between abdominal symptoms and QOL is well-documented, and we anticipated that a comparison of QOL between NSAID and non-NSAID ulcer patients would confirm that there is no real difference in symptoms between these two groups. However, physical QOL was actually lower in the NSAID ulcer patients than in the non-NSAID ulcer patients. This reduction in physical QOL may not necessarily have been caused by peptic ulcer; it could instead be an effect of the underlying disease for which NSAIDs were used. Within the category of QOL scores, a reduction in the physical summary score alone could indicate the influence of underlying disease, while no difference in the psychological summary score suggests that symptoms do not differ between the two groups. In fact, 70% of patients who used NSAIDs in this research had conditions such as osteoarthritis and spinal stenosis that negatively affected their activities of daily living, and the results described above may have been affected by those conditions.

Why might symptoms not be reduced, even if the patient is using NSAIDs? Symptom severity is experienced differently from person to person, so this question cannot be answered accurately except by interviewing the same patients about the symptoms of ulcers of similar severity that developed on two different occasions: once while taking NSAIDs and the other while not taking NSAIDs. It seems possible that NSAIDs would inhibit symptoms to some extent at ulcer onset, but that inhibition was too small to be detected by the present recall-based research method. However, the present research results clearly contradict the common wisdom that NSAID-induced peptic ulcers tend to be asymptomatic at onset. It is possible that NSAIDs do not have much effect on abdominal symptoms. This is because the most common reason for the discontinuation of NSAIDs is that patients experience upper abdominal symptoms. Oral NSAIDs do not only directly elicit a mucous membrane disorder but oral administration also accelerates the secretion of gastric acid, which is one of the reasons for symptom onset.

This study has several limitations. First, it was an observational study. Second, a relatively large number of patients (26 patients) were excluded from consideration because they provided incomplete or insufficient information in the questionnaire. The patients were excluded from this study if they provided incomplete or insufficient entries for even one item. This large number of exclusions could be due to the research design, which included small general hospitals, as well as large flagship hospitals. Most of the incomplete or insufficient questionnaires came from the general hospitals, probably due to a relative lack of awareness of the principles of clinical research at such hospitals. In addition, regarding the *H. pylori* infection, we did not check it in the present study, because we have focused our attention on the effects of NSAID and/or LDA use on gastrointestinal symptoms when ulcers developed at our clinic or in a related facility.

In conclusion, this was the first observational study to examine and compare symptoms and QOL at ulcer onset between NSAID/LDA ulcers and non-NSAID/LDA ulcers in patients in the acute stage of peptic ulcer. The severity of upper abdominal symptoms at peptic ulcer onset was similar between NSAID and non-NSAID users.

## Figures and Tables

**Table 1:** Characteristics of patients.

	None (*n* = 85)	NSAIDs (*n* = 52)	LDA (*n* = 21)	NSAIDs/LDA (*n* = 16)
Mean age (±SD) (years)	60.6 ± 17.0	64.4 ± 15.0	73.7 ± 11	71.3 ± 11.2
Male/female	56/29	24/28	16/5	9/7
Alcohol drinker (%)	66 (77.6%)	52 (100%)	21 (100%)	16 (100%)
Smoker (%)	66 (77.6%)	15 (28.8%)	5 (23.8%)	6 (37.5%)
Regular use of NSAIDs (>1 month)	—	15 (28.8%)	—	8 (50%)
Regular use of LDA (>1 month)	—	—	17 (81.0%)	13 (81.3%)
Antiulcer drug	41 (48.2%)	39 (75%)	13 (61.9%)	15 (93.8%)
Medication of PPI	14 (16.5%)	10 (19.2%)	2 (9.5%)	5 (31.3%)

NSAIDs: nonsteroidal anti-inflammatory drugs; LDA: low-dose aspirin; PPI: proton-pump inhibitor.
